# Six-month immune responses to mRNA-1273 vaccine in combination antiretroviral therapy treated late presenter people with HIV according to previous SARS-CoV-2 infection

**DOI:** 10.1097/QAD.0000000000003585

**Published:** 2023-05-18

**Authors:** Matteo Augello, Valeria Bono, Roberta Rovito, Camilla Tincati, Antonella d’Arminio Monforte, Giulia Marchetti

**Affiliations:** Clinic of Infectious Diseases and Tropical Medicine, San Paolo Hospital, ASST Santi Paolo e Carlo, Department of Health Sciences, University of Milan, Milan, Italy.

**Keywords:** coronavirus disease 2019, HIV/AIDS, immune responses, SARS-CoV-2, vaccines

## Abstract

Immune responses to severe acute respiratory syndrome coronavirus 2 (SARS-CoV-2) mRNA vaccines in people with HIV (PWH) with a history of late presentation (LP) and their durability have not been fully characterized.

**Design:**

In this prospective, longitudinal study, we sought to assess T-cell and humoral responses to SARS-CoV-2 mRNA vaccination up to 6 months in LP-PWH on effective combination antiretroviral therapy (cART) as compared to HIV-negative healthcare workers (HCWs), and to evaluate whether previous SARS-CoV-2 infection modulates immune responses to vaccine.

**Methods:**

SARS-CoV-2 spike (S)-specific T-cell responses were determined by two complementary flow cytometry methodologies, namely activation-induced marker (AIM) assay and intracellular cytokine staining (ICS), whereas humoral responses were measured by ELISA [anti-receptor binding domain (RBD) antibodies) and receptor-binding inhibition assay (spike-ACE2 binding inhibition activity), before vaccination (T0), 1 month (T1) and 5 months (T2) after the second dose.

**Results:**

LP-PWH showed at T1 and T2 significant increase of: S-specific memory and circulating T follicular helper (cTfh) CD4^+^ T cells; polyfunctional Th1-cytokine (IFN-γ, TNF-α, IL-2)- and Th2-cytokine (IL-4)-producing S-specific CD4^+^ T cells; anti-RBD antibodies and spike-ACE2 binding inhibition activity. Immune responses to vaccine in LP-PWH were not inferior to HCWs overall, yet S-specific CD8^+^ T cells and spike-ACE2 binding inhibition activity correlated negatively with markers of immune recovery on cART. Interestingly, natural SARS-CoV-2 infection, while able to sustain S-specific antibody response, seems less efficacious in inducing a T-cell memory and in boosting immune responses to vaccine, possibly reflecting an enduring partial immunodeficiency.

**Conclusions:**

Altogether, these findings support the need for additional vaccine doses in PWH with a history of advanced immune depression and poor immune recovery on effective cART.

## Introduction

The incidence of coronavirus disease 2019 (COVID-19) among people with HIV (PWH) has been reported similar [1–3] or even lower [4–6] than in the general population, suggesting that HIV *per se* is not a risk factor for severe acute respiratory syndrome coronavirus 2 (SARS-CoV-2) infection. Likewise, studies evaluating COVID-19 outcome in PWH have yielded conflicting results due to multiple confounding factors [7,8], displaying similar [9,10] or higher [3,11–13] disease severity and mortality compared to people without HIV.

Data on the efficiency of immune response to SARS-CoV-2 in PWH are controversial [14]. T-cell and humoral responses after SARS-CoV-2 infection have been reported not inferior to those in HIV-negative peers in two previous studies [15,16], and yet related to CD4^+^/CD8^+^ ratio [15]. Conversely, others found lower and more exhausted SARS-CoV-2–specific T cells [17–19], or reduced humoral responses [6] in PWH recovered from COVID-19, especially in those with detectable HIV viremia and low CD4^+^ T-cell counts. Furthermore, in a murine model of acute SARS-CoV-2 infection, the depletion of CD4^+^ T cells led to diminished antibodies response and delayed viral clearance [20], highlighting the paramount role of CD4^+^ T cells in controlling viral replication and regulating SARS-CoV-2–specific immune responses.

The observation that PWH have lower responses to hepatitis B virus vaccination [21,22], show more rapid wane of neutralizing antibodies after yellow fever vaccination [23], and mount variable humoral responses to other vaccines depending on CD4^+^ T-cell counts [24], raise the concern that PWH, especially those with incomplete immune reconstitution despite virally effective combination antiretroviral therapy (cART), may not adequately respond to SARS-CoV-2 vaccines.

Despite such concerns, several studies have recently shown that immune responses to mRNA vaccines in PWH with optimal immunological response are comparable to those in healthy individuals but may be reduced in PWH with poor CD4^+^ T-cell recovery [25–31]. Likewise, vaccine effectiveness, namely the ability of vaccine to protect against symptomatic illness and hospitalization/death, has been proven among a large population-based cohort of PWH with well controlled HIV infection after two doses of SARS-CoV-2 vaccine [32], yet it remains to be determined in PWH who have moderate to severe immunodeficiency.

However, studies carried out so far mainly evaluated humoral immunogenicity, overlooking magnitude and functionality of T-cell responses [25–28,30,31]. T cells have been described to play a pivotal role in vaccine-induced protection as they defend against severe disease and hospitalization, while neutralizing antibodies primarily protect against acquisition of infection [33–41]. Of note, T-cell dysfunction has been long acknowledged as a hallmark of HIV infection [42], particularly in people with a history of late presentation [43–48] and poor immunological recovery [49–56], raising the concern that these populations may not mount adequate T-cell responses to SARS-CoV-2 vaccines.

Additionally, while COVID-19–recovered individuals have been proven to mount stronger immune responses to SARS-CoV-2 vaccines compared to virus-naive ones in the general population [57–59], whether previous SARS-CoV-2 infection may modulate T-cell and humoral responses to such vaccines in PWH is still a matter of debate.

Hence, in this study, we sought to longitudinally investigate the magnitude and the quality of T-cell and humoral responses to mRNA-1273 vaccine up to 6 months apart from the primary cycle in successfully cART-treated PWH who had started therapy as late presenters (LP-PWH), compared to HIV-negative peers. We also aimed to evaluate the role of previous SARS-CoV-2 infection in conditioning immune responses to vaccine in this population.

## Methods

### Study design

In this prospective, longitudinal study, we consecutively enrolled LP-PWH (CD4^+^ T-cell *nadir* < 350 cells/μl and/or history of AIDS-defining events) on virologically effective cART (HIV-RNA < 20 copies/ml), who received mRNA-1273 vaccine (two doses 28 days apart) at the Clinic of Infectious Diseases and Tropical Medicine, ASST Santi Paolo e Carlo, Department of Health Sciences, University of Milan, Milan, Italy. HIV-negative healthcare workers (HCWs), who received BNT162b2 (two doses 21 days apart) were also consecutively enrolled as controls.

Peripheral blood samples were collected in EDTA tubes from all study participants before vaccination (T0), 1 month and 5 months after the second dose (T1 and T2, respectively) (Figure S1, Supplemental Digital Content). Plasma was separated by centrifugation and stored at –80°C. Peripheral blood mononuclear cells (PBMCs) were obtained by Ficoll density gradient centrifugation and stored at –80°C and then in liquid nitrogen.

Demographic and clinical characteristics of the study population as well as HIV-related features of LP-PWH were also collected.

The study was approved by the Institutional Ethics Committee and written informed consent was obtained from each participant. All research was performed in accordance with the Declaration of Helsinki.

### SARS-CoV-2-specific T cells

SARS-CoV-2-specific T cells were determined within PBMCs by means of two complementary flow cytometry methodologies, i.e. activation-induced marker (AIM) assay and intracellular cytokine staining (ICS) [60,61] (Figure S2, Supplemental Digital Content).

Briefly, 1.5 × 10^6^ thawed PBMCs were plated in complete RPMI containing 10% human serum supplemented with 1% penicillin–streptomycin–glutamine. Overnight-rested PBMCs were stimulated for 20 h with a pool of 15-mer peptides (1 μg/ml) covering the immunodominant sequence domain of the wild-type spike (S) protein (PepTivator SARS-CoV-2, Miltenyi Biotec). *Staphylococcus* enterotoxin B (SEB, Sigma-Aldrich) (1 μg/ml) was used as positive control, while negative controls were left untreated.

For AIM assay, PBMCs were washed in FACS buffer (PBS with 2% BSA) and stained with the appropriate surface antibodies for 20 min at 4°C in the dark, fixed with 2% paraformaldehyde (PFA) for 30 min at 4°C, washed, and resuspended in 500 μl of phosphate buffered saline (PBS). Dead cells were labeled using Viobility Fixable Dye (Miltenyi Biotec). Antibodies used were: CD4–APC-Vio770, CD8–PerCP-Vio700, CXCR5–APC (Miltenyi Biotec), CD45RA–BV421, CCR7–PE, CD69–FITC (BD Biosciences), and CD137–PeCy-7 (BioLegend).

For ICS assay, brefeldin A (1 mg/ml) was added after 1 h of stimulation. Cells were harvested and stained for surface markers for 20 min at 4°C in the dark; after 2% PFA fixation, cells were permeabilized with 0.2% saponin and stained for intracellular cytokines for 30 min at room temperature. Dead cells were labeled using Viobility Fixable Dye (Miltenyi Biotec). Antibodies used were: CD4–APC-Vio770, CD8–PerCP-Vio700, IL-2–APC (Miltenyi Biotec), IL-17A–PE, IL-4–FITC, TNF-α–VioBlue, IFN-γ–PE-Vio770 (BD Biosciences).

Samples were acquired using FACSVerse cytometer (BD Biosciences) and FCS files were analyzed using FlowJo 10.7.2 (BD Biosciences).

T-cell subsets were defined as: CCR7+CD45RA+ (naïve, N), CCR7+CD45RA– (central memory, CM), CCR7–CD45RA– (effector memory, EM), CCR7–CD45RA+ (effector memory re-expressing CD45RA, EMRA), CD4+CXCR5+ (circulating T follicular helper, cTfh), CD8+CXCR5+ (circulating T follicular cytotoxic, cTfc).

SARS-CoV-2-specific T cells were measured subtracting unspecific background activation (AIM) or cytokine-production (ICS) in paired unstimulated control samples from stimulated samples; negative values were set to zero. By AIM assay, frequencies (percentage, %) of CD69+CD137+ [58,60] within total, CM, EM, EMRA, and cTfh/cTfc CD4^+^ and CD8^+^ T cells were measured. By ICS assay, cytokine (IFN-γ, TNF-α, IL-2, IL-4, IL-17A)-producing T cells were determined and expressed as both frequency (percentage, % of CD4^+^ and CD8^+^ T cells) and integrated median fluorescence intensity (iMFI, obtained by multiplying the frequency by the MFI for each cytokine-producing subset). T-cell polyfunctionality was assessed by using the FlowJo Boolean Gating tool and SPICE 6.0 to identify single-, dual-, triple- cytokine-producing SARS-CoV-2-specific Th1 cells.

### Total anti-receptor binding domain antibodies

Total anti-receptor binding domain (anti-RBD) antibodies were determined in plasma samples by a homemade ELISA as previously described [62]. Briefly, high-binding 96-well plates (Greiner Bio-One) were coated with 3 μg/ml of recombinant wild-type SARS-CoV-2 receptor binding domain (RBD) (ACROBiosystems) diluted in 0.5 mmol/l of carbonate-bicarbonate buffer pH 9.6 (Sigma-Aldrich) and incubated overnight at 4°C. Plates were washed with PBS–0.05% Tween-20 and blocked for 1 h with PBS–2% BSA at 37°C. Plasma samples were serially diluted in PBS–1% BSA in triplicates (1:40, 1:240 and 1:1440), added to plates, and incubated for 2 h at 37°C. A mix of biotinylated goat antihuman k and λ light chain were used at 1:2500 (Bethyl Laboratories, Inc., A80–115B and A80–116B) for detection, followed by avidin-HRP diluted at 1:2000 (ThermoFischer Scientific), for 30 min at room temperature in the dark and mild agitation. The detection was carried out with 3,3’,5,5’-tetramethylbenzidine (TMB) (Invitrogen) and quenched with 1 mol/l H_2_SO_4_. Two plasma samples collected before the SARS-CoV-2 pandemic were included as negative controls, whereas an RBD-specific monoclonal antibody (Human Anti-SARS-CoV-2 Spike RBD Monoclonal Antibody, clone BIB116, Creative Diagnostics) was included as positive control. The optical density (OD) was measured by using EnSight (Multimode Plate Reader, PerkinElmer) at 450 and 620 nm, and the area under the curve (AUC) was determined with GraphPad Prism 9.2.

### Receptor-binding inhibition assay

A receptor-binding inhibition assay, based on antibody-mediated blockage of ACE2-Spike RBD interaction, was employed to measure plasma spike-ACE2 binding inhibition (spike-blocking) activity, which is a surrogate of neutralization activity, as previously described [63,64]. Briefly, high-binding 96-well plates (Corning) were coated with 2 μg/ml of recombinant human ACE2-Fc (InvivoGen) diluted in 100 mmol/l carbonate–bicarbonate buffer pH 9.6 (Sigma-Aldrich) and incubated overnight at 4°C. Plates were washed with PBS–0.05% Tween-20 and blocked with PBS–2% BSA for 1 h at room temperature. Plasma samples were diluted 1:80 in triplicates in PBS–1% BSA and incubated with 12 ng of recombinant wild-type SARS-CoV-2 RBD-HRP (ACROBiosystems) for 1 h at 37°C. Plates were washed and incubated with the preincubated plasma and RBD-HRP for 1 h at room temperature, then detected with TMB and 1 mol/l H_2_SO_4_. RBD-HRP alone and plasma with no RBD-HRP were used as controls. The OD was measured by using EnSight (Multimode Plate Reader, PerkinElmer) at 450 and 570 nm. The results were expressed as percentage (%) of inhibition, calculated as [(1 – sample OD)/average negative control OD)] × 100.

### Statistical analyses

Wilcoxon signed-rank test was used for longitudinal analyses to assess immune responses to vaccine at T1 and T2 vs. T0. Mann–Whitney *U* test was used for cross-sectional analyses to compare study groups. Spearman's correlation test was used to correlate immune status and immune responses to vaccine in LP-PWH. Data were analyzed and graphed with GraphPad Prism 9.2.0. Permutation test in SPICE 6.0 was employed to compare polyfunctionality patterns of SARS-CoV-2-specific cytokine-producing Th1 cells in the two groups. *P* values <0.05 were considered statistically significant.

## Results

### Study population

Twenty LP-PWH and 20 HCWs were enrolled. In each study group, 10 (50%) participants had a history of previous SARS-CoV-2 infection (SARS-CoV-2-experienced/recovered) and 10 (50%) did not (SARS-CoV-2–naive) (Figure S1, Supplemental Digital Content).

Demographic and clinical characteristics of the study population as well as HIV-related features of LP-PWH are summarized in Table 1.

**Table 1:** Demographic and clinical features of the study population and HIV-related characteristics of LP-PWH.

	LP-PWH (*n* = 20)	HCWs (*n* = 20)	*P* value LP-PWH vs. HCWs^∗^
Age, years, median (IQR)	57 (47–62)	55 (45–61)	0.3500
Sex, *n* (%)			0.1908
Male	15 (75)	10 (50)	
Female	5 (25)	10 (50)	
Ethnicity, *n* (%)			0.1264
Caucasian	15 (75)	20 (100)	
Latin-American	3 (15)	0 (0)	
Afro-American	1 (5)	0 (0)	
African	1 (5)	0 (0)	
Comorbidities, *n* (%)			
Hypertension	5 (25)	2 (10)	0.4075
Chronic heart disease	2 (10)	0 (0)	0.4872
Myocardial infarction	1 (5)	0 (0)	>0.9999
Peripheral vascular disease	1 (5)	0 (0)	>0.9999
Chronic pulmonary disease	2 (10)	2 (10)	>0.9999
Chronic kidney disease	2 (10)	1 (5)	>0.9999
Liver disease	3 (15)	0 (0)	0.2308
Diabetes	3 (15)	0 (0)	0.2308
Charlson comorbidity index°, median (IQR)	2 (0–4)	1 (0–2)	0.1153
BMI, *n* (%)			0.2196
<25 kg/m^2^	7 (35)	8 (40)	
25–30 kg/m^2^	8 (40)	7 (35)	
>30 kg/m^2^	3 (15)	0 (0)	
Unknown	2 (10)	5 (25)	
Smoking, *n* (%)			0.1353
Yes	5 (25)	3 (15)	
No	9 (45)	15 (75)	
Unknown	6 (30)	2 (10)	
			
*HIV-related characteristics*			
Epidemiology, *n* (%)			
MSM	8 (40)	NA	NA
IDU	2 (10)	NA	NA
Other	10 (50)	NA	NA
Viro-immunologic parameters, median (IQR)			
CD4^+^*nadir*, cells/μl	67 (32–215)	NA	NA
HIV-RNA *zenith*, copies/ml	60 816 (22 402–242 511)	NA	NA
Current %CD4^+^	25 (12–30)	NA	NA
Current CD4^+^, cells/μl	404 (192–615)	NA	NA
Current %CD8^+^	46 (35–60)	NA	NA
Current CD8^+^, cells/μl	809 (590–1008)	NA	NA
Current CD4^+^/CD8^+^ ratio	0.57 (0.20–0.87)	NA	NA
Current HIV-RNA, copies/ml	<20	NA	NA
Current CD4^+^, *n* (%)			
<350 cells/μl	8 (40)	NA	NA
350–500 cells/μl	5 (25)	NA	NA
>500 cells/μl	7 (35)	NA	NA
Previous AIDS diagnosis, *n* (%)	8 (40)	NA	NA
Time from HIV diagnosis, years, median (IQR)	14.5 (6–25)	NA	NA
Current cART regimen, *n* (%)			
INSTI-based triple	11 (55)	NA	NA
INSTI-based dual	6 (30)	NA	NA
NNRTI-based triple	3 (15)	NA	NA
Time from HIV diagnosis to cART initiation, days, median (IQR)	50 (18–2166)	NA	NA
Duration of cART, years, median (IQR)	12 (6–17)	NA	NA

LP-PWH, late presenter people with HIV; BMI, body mass index; cART, combination antiretroviral therapy; HCWs, healthcare workers; IDU, injective drugs use; INSTI, integrase strand transfer inhibitor; IQR, interquartile range; MSM, men who have sex with men; NA, not applicable; NNRTI, nonnucleoside reverse transcriptase inhibitor; °age-adjusted.

∗ *Statistical analyses*, Mann–Whitney *U* test, Fisher exact test, chi-square test, as appropriate.

No significant differences in age, sex, ethnicity, comorbidities, body mass index (BMI), and smoking habit were registered between the two groups.

Eight (40%) LP-PWH had a history of AIDS-defining conditions. All LP-PWH were on long-term [median: 12 (IQR: 6–17) years] virologically effective (HIV-RNA < 20 copies/ml) cART, with various grades of immune recovery [median CD4^+^ T-cell count: 404 (192–615) cells/μl; CD4^+^ T-cell percentage: 25 (12–30)%; CD4^+^/CD8^+^ ratio: 0.57 (0.20–0.87)]. As per definition, median CD4^+^ T-cell *nadir* in LP-PWH was 67 (32–215) cells/μl.

As expected, LP-PWH showed lower CD4^+^ T cells (*P* < 0.0001), yet higher CD8^+^ T cells (*P* < 0.0001) as compared to HCWs at baseline (T0) (Figure S3, Supplemental Digital Content). Regarding immune phenotypes, N CD4^+^ (*P* = 0.0025) and CD8^+^ T cells (*P* = 0.0003) were lower, while EM CD4^+^ (*P* = 0.0003) and CD8^+^ T cells (*P* < 0.0001) were higher in LP-PWH. Furthermore, a greater percentage of cTfc CD8^+^ T cell was observed in LP-PWH (*P* = 0.0056) (Figure S3, Supplemental Digital Content).

### Vaccine-induced SARS-CoV-2-specific T-cell responses

**Fig. 1:**
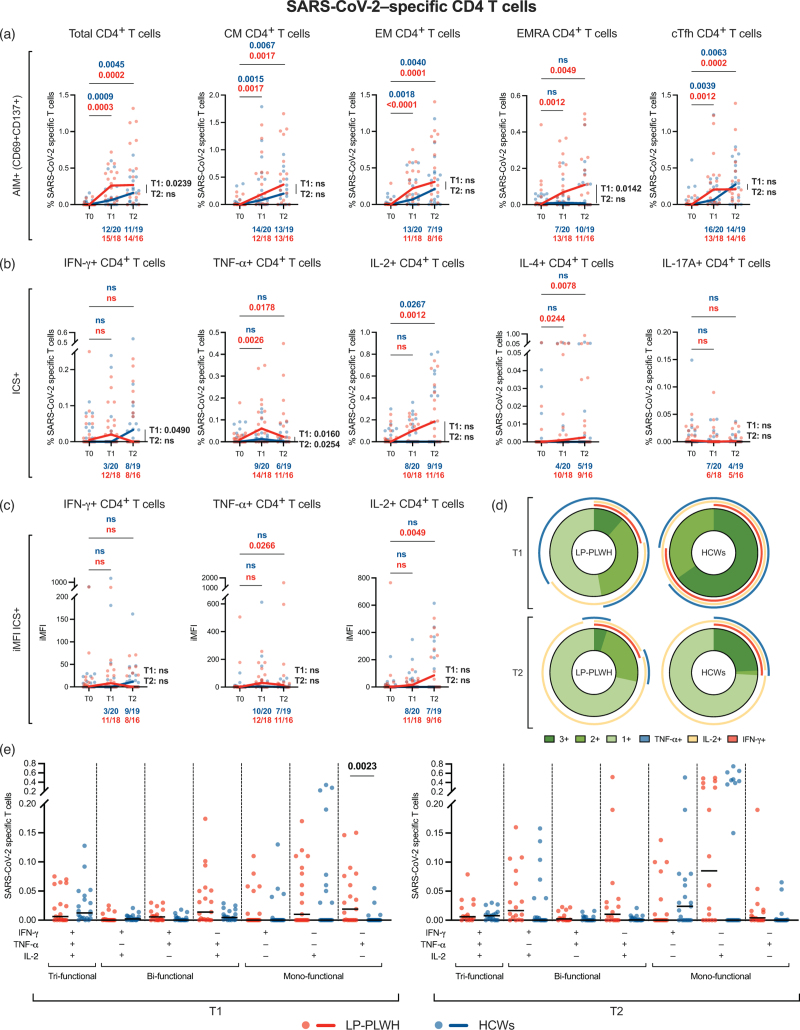
Vaccine-induced SARS-CoV-2-specific CD4^+^ T-cell responses.

LP-PWH showed expansion of SARS-CoV-2–specific total (*P*_T0–T1_ = 0.0003, *P*_T0–T2_ = 0.0002), CM (*P*_T0–T1_ = 0.0017, *P*_T0–T2_ = 0.0017), EM (*P*_T0–T1_ < 0.0001, *P*_T0–T2_ = 0.0001), EMRA (*P*_T0–T1_ = 0.0012, *P*_T0–T2_ = 0.0049), and cTfh (*P*_T0–T1_ = 0.0012, *P*_T0–T2_ = 0.0002) CD4^+^ T cells at both time points as compared to baseline (Fig. 1a). Likewise, HCWs displayed rise of SARS-CoV-2-specific total (*P*_T0–T1_ = 0.0009, *P*_T0–T2_ = 0.0045), CM (*P*_T0–T1_ = 0.0015, *P*_T0–T2_ = 0.0067), EM (*P*_T0–T1_ = 0.0018, *P*_T0–T2_ = 0.0040), and cTfh (*P*_T0–T1_ = 0.0039, *P*_T0–T2_ = 0.0063) at T1 and T2, but not EMRA CD4^+^ T cells (Fig. 1a). When comparing the two study groups at T1 and T2, vaccine-induced SARS-CoV-2–specific memory and cTfh CD4^+^ T cells in LP-PWH were not inferior to HCWs, with the latter showing lower S-specific total (*P* = 0.0239) and EMRA CD4^+^ T cells (*P* = 0.0142) at T1 (Fig. 1a).

(a) Frequency (percentage, %) of SARS-CoV-2-specific T cells within total, CM, EM, EMRA, and cTfh CD4^+^ T cells, as determined by AIM assay (CD69+CD137+), in LP-PWH and HCWs at T0, T1, and T2. (b) Frequency of cytokine (IFN-γ, TNF-α, IL-2, IL-4, IL-17A)-producing SARS-CoV-2-specific T cells within total CD4^+^ T cells, as determined by ICS assay, in LP-PWH and HCWs at T0, T1, and T2. (c) iMFI of Th1-cytokine (IFN-γ, TNF-α, IL-2)-producing SARS-CoV-2-specific T cells within CD4^+^ T cells, as determined by ICS assay, in LP-PWH and HCWs at T0, T1, and T2. (d) Donut charts showing the median distribution of polyfunctionality profiles in SARS-CoV-2-specific cytokine-producing Th1 cells of LP-PWH and HCWs at T1 and T2. The donut slices represent median percentages of tri- (3+), bi- (2+), and mono- (1+) functional T cells. The arches around the circumference indicate the particular cytokine (IFN-γ, TNF-α, or IL-2) produced by the portion of T cells that lie under the arc; parts of the donut surrounded by multiple arches represent polyfunctional cells. (e) Frequency of tri-, bi-, and mono-functional SARS-CoV-2-specific Th1 cells within total CD4^+^ T cells in LP-PWH and HCWs at T1 and T2. *Red/blue dots*: individual values; *black bars*: medians; *red/blue lines* connecting median values at T0, T1, and T2; *fractions below plots*: number of individuals out of the total analyzed which are above their individual baseline at each time point; *longitudinal analyses* (T1 and T2 compared to T0): Wilcoxon signed-rank test (*P* values in red/blue); *cross-sectional analyses* (at each time point): Mann–Whitney *U* test (*P* values in black); *analyses between donut charts*: SPICE permutation test. SARS-CoV-2-specific T cells were measured subtracting unspecific background activation (AIM) or cytokine-production (ICS) in paired unstimulated control samples from stimulated samples.

**Fig. 2:**
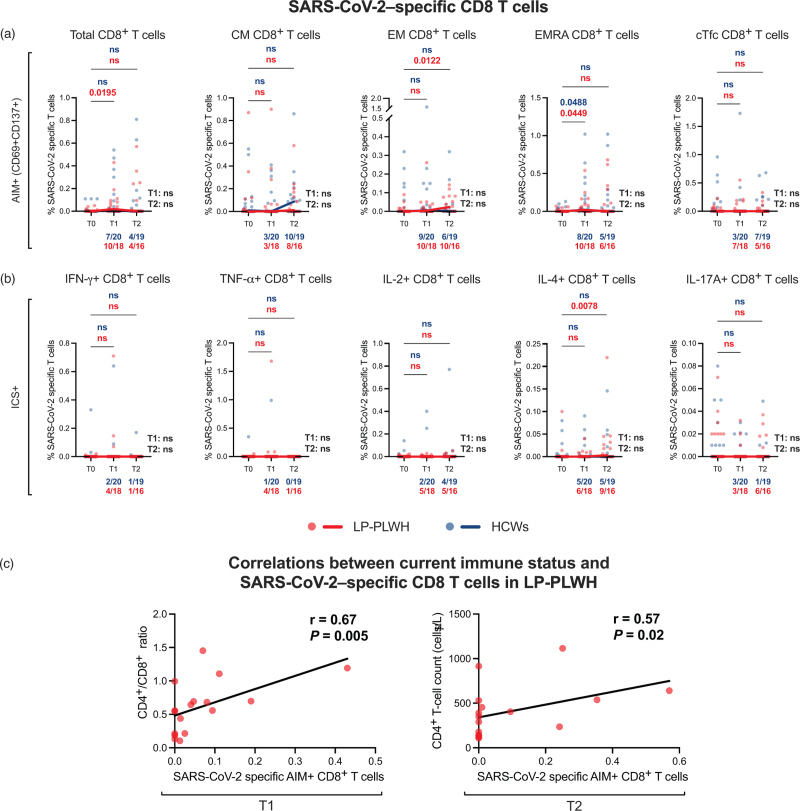
Vaccine-induced SARS-CoV-2–specific CD8^+^ T-cell responses.

Overall, CD8^+^ T-cell responses were lower than CD4^+^ T-cell responses (Figure S4A, Supplemental Digital Content), with a significant expansion of SARS-CoV-2–specific total (*P*_T0–T1_ = 0.0195) and EMRA (*P*_T0–T1_ = 0.0449) CD8^+^ T cells at T1 and EM CD8^+^ T cells (*P*_T0–T2_ = 0.0122) at T2 in LP-PWH, as well as an increase of EMRA CD8^+^ T cells (*P*_T0–T1_ = 0.0488) at T1 in HCWs, with no differences in frequencies of S-specific CD8^+^ T cells between the two groups (Fig. 2a).

(a) Frequency (percentage, %) of SARS-CoV-2-specific T cells within total, CM, EM, EMRA, and cTfc CD8^+^ T cells, as determined by AIM assay (CD69+CD137+), in LP-PWH and HCWs at T0, T1, and T2. (b) Frequency of cytokine (IFN-γ, TNF-α, IL-2, IL-4, IL-17A)-producing SARS-CoV-2–specific T cells within total CD8^+^ T-cells, as determined by ICS assay, in LP-PWH and HCWs at T0, T1, and T2. *Red/blue dots*: individual values; *red/blue lines* connecting median values at T0, T1, and T2; *fractions below plots*: number of individuals out of the total analyzed which are above their individual baseline at each time point; *longitudinal analyses* (T1 and T2 compared to T0): Wilcoxon signed-rank test (*P* values in red/blue); *cross-sectional analyses* (at T1 and T2): Mann–Whitney U test (*P* values in black). SARS-CoV-2–specific T cells were measured subtracting unspecific background activation (AIM) or cytokine-production (ICS) in paired unstimulated control samples from stimulated samples. (c) Correlations between current immune status (CD4^+^/CD8^+^ ratio and CD4^+^ T-cell count) and SARS-CoV-2-specific CD8^+^ T cells in LP-PWH at T1 and T2, respectively. *Red dots*: individual values; *black line*: simple linear regression line; *analysis*: Spearman's correlation test.

When assessing cytokine-producing SARS-CoV-2–specific T cells, LP-PWH displayed a significant increase in frequency of TNF-α+ and IL-4+ CD4^+^ T cells at both time points (TNF-α+: *P*_T0-T1_ = 0.0026, *P*_T0-T2_ = 0.0178; IL-4+: *P*_T0-T1_ = 0.0244, *P*_T0–T2_ = 0.0078), and IL-2+ CD4^+^ T cells at T2 (*P*_T0–T2_ = 0.0012) (Fig. 1b). HCWs only showed an expansion of IL-2+ CD4^+^ T-cells at T2 (*P*_T0–T2_ = 0.0267) (Fig. 1b). Overall, LP-PWH exhibited higher frequencies of S-specific IFN-γ+ CD4^+^ T cells at T1 (*P* = 0.049) and TNF-α+ CD4^+^ T cells at both time points (*P*_T1_ = 0.016, *P*_T2_ = 0.0254) (Fig. 1b). By iMFI, LP-PWH presented a rise of SARS-CoV-2-specific TNF-α+ (*P*_T0–T2_ = 0.0266) and IL-2+ (*P*_T0–T2_ = 0.0049) CD4^+^ T cells at T2 (Fig. 1c), with no overall differences in S-specific cytokine-producing CD4^+^ T cells between the two study groups at both time points (Fig. 1c).

Next, we assessed polyfunctionality of vaccine-induced SARS-CoV-2-specific CD4^+^ T cells, as multifunctional Th1 cells (IFN-γ+TNF-α+IL-2+) have been described to provide a better correlate of immune protection against infection after vaccination [65]. LP-PWH and HCWs displayed comparable median distribution of polyfunctionality profiles within SARS-CoV-2-specific cytokine-producing Th1 cells at both time points, with a nonsignificant trend towards higher relative frequencies of tri-functional S-specific Th1 cells in HCWs (Fig. 1d). Frequencies of mono-functional, bi-functional and tri-functional S-specific Th1 cells within total CD4 T cells were comparable between LP-PWH and HCWs, except for higher percentages of mono-functional TNF-α+ IFN-γ–IL-2– CD4^+^ T cells in LP-PWH at T1 (*P* = 0.0023) (Fig. 1e).

CD8^+^ T-cell responses confirmed inferior to CD4^+^ T-cell responses also by evaluating intracellular cytokines production (Figure S4B, Supplemental Digital Content). Only a significant expansion in frequencies of SARS-CoV-2-specific IL-4+ CD8^+^ T cells at T2 in LP-PWH (*P* = 0.0078) was detected, with no differences in S-specific cytokine-producing CD8^+^ T cells between the two groups (Fig. 2b).

Interestingly, total AIM+ SARS-CoV-2–specific CD8^+^ T cells were found to correlate positively with CD4^+^/CD8^+^ ratio at T1 (*r* = 0.67, *P* = 0.005) and with CD4^+^ T-cell count at T2 (*r* = 0.57, *P* = 0.02) in LP-PWH (Fig. 2c).

### Vaccine-induced SARS-CoV-2-specific humoral responses

**Fig. 3:**
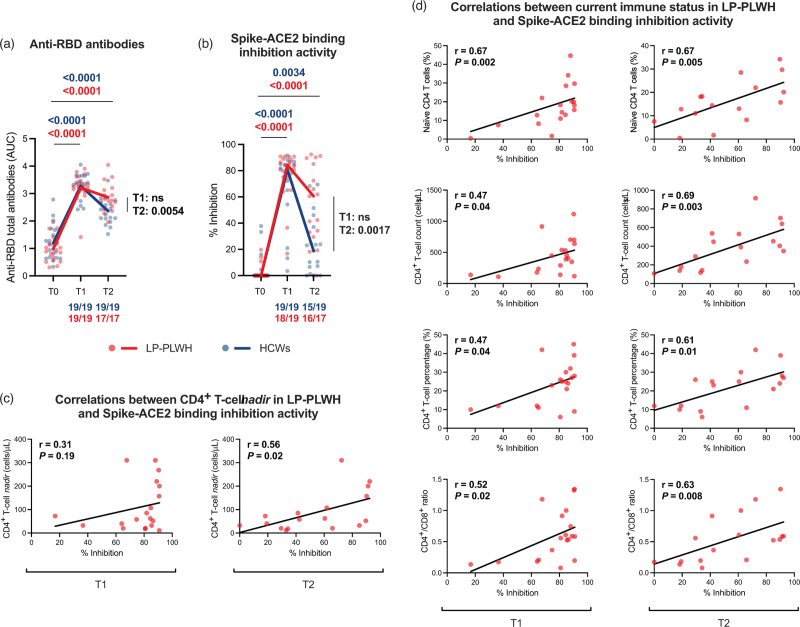
Vaccine-induced SARS-CoV-2-specific humoral responses.

Total anti-RBD antibodies increased at T1 and T2 as compared to baseline in both LP-PWH (*P*_T0–T1_ < 0.0001, *P*_T0–T2_ < 0.0001) and HCWs (*P*_T0–T1_ < 0.0001, *P*_T0–T2_ < 0.0001) (Fig. 3a). When comparing LP-PWH and HCWs, no differences were observed at T1, but higher levels of anti-RBD antibodies were detected in LP-PWH at T2 (*P* = 0.0054) (Fig. 3a).

(a) Anti-RBD total antibodies expressed as AUC in LP-PWH and HCWs at T0, T1, and T2. (b) Spike-ACE2 binding inhibition activity expressed as percentage (%) of inhibition of ACE2-spike RBD interaction in LP-PWH and HCWs at T0, T1, and T2. *Red/blue dots*: individual values; *red/blue lines* connecting median values at T0, T1, and T2; *fractions below plots*: number of individuals out of the total analyzed which are above their individual baseline at each time point; *longitudinal analyses* (T1 and T2 compared to T0): Wilcoxon signed-rank test (*P* values in red/blue); *cross-sectional analyses* (at T1 and T2): Mann–Whitney *U* test (*P* values in black). (c) Correlations between CD4^+^ T-cell *nadir* and Spike-ACE2 binding inhibition activity in LP-PWH at T1 and T2. (d) Correlations between current immune status (naïve CD4^+^ T cells, CD4^+^ T-cell count, CD4^+^ T-cell percentage, CD4/CD8 ratio) and spike-ACE2 binding inhibition activity in LP-PWH at T1 and T2. *Red dots*: individual values; *black line*: simple linear regression line; *analysis*: Spearman's correlation test.

Furthermore, a rise of spike-ACE2 binding inhibition activity at both time points was detected in LP-PWH (*P*_T0–T1_ < 0.0001, *P*_T0–T2_ < 0.0001) and HCWs (*P*_T0–T1_ < 0.0001, *P*_T0–T2_ = 0.0021) (Fig. 3b). As for anti-RBD antibodies, no significant differences between groups were registered at T1, yet LP-PWH showed higher spike-blocking activity at T2 than HCWs (*P* = 0.0017) (Fig. 3b). Interestingly, in LP-PWH, spike-ACE2 binding inhibition activity was found to positively correlate with CD4^+^ T-cell *nadir* [T1: *r* = 0.31, *P* = 0.19; T2: *r* = 0.56, *P* = 0.02] (Fig. 3c), as well as current immune status, i.e. naïve CD4^+^ T cells [T1: *r* = 0.67, *P* = 0.002; T2: *r* = 0.67, *P* = 0.005], CD4^+^ T-cell count [T1: *r* = 0.47, *P* = 0.04; T2: *r* = 0.69, *P* = 0.003], CD4^+^ T-cell percentage [T1: *r* = 0.47, *P* = 0.04; T2: *r* = 0.61, *P* = 0.01], and CD4^+^/CD8^+^ ratio [T1: *r* = 0.52, *P* = 0.02; T2: *r* = 0.63, *P* = 0.008] (Fig. 3d).

No correlations were found between T-cell and humoral responses to vaccine in both study groups (data not shown).

### SARS-CoV-2-specific T-cell and humoral responses in SARS-CoV-2-naive vs. -experienced vaccinees

**Fig. 4:**
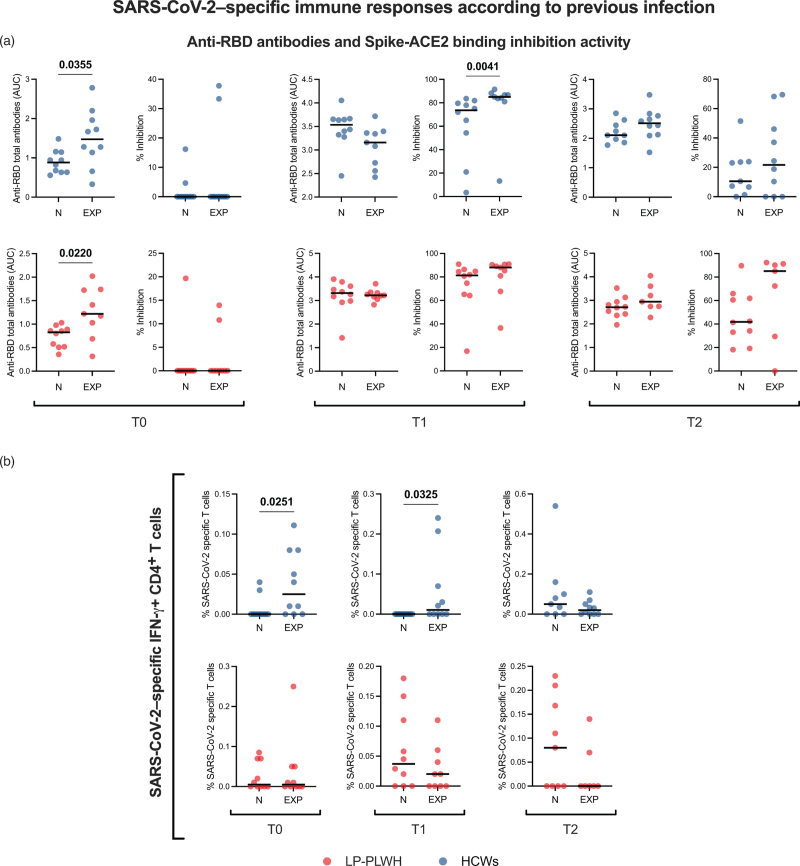
SARS-CoV-2-specific immune responses according to previous infection.

When assessing vaccine-elicited immune responses according to previous COVID-19, we found that SARS-CoV-2-experienced HCWs showed higher anti-RBD antibodies as compared to naïve individuals only prior to vaccine administration (*P* = 0.0355); after vaccination, they displayed greater Spike-blocking activity at T1 (*P* = 0.0041), but not at T2 (Fig. 4a). SARS-CoV-2-recovered LP-PWH showed higher anti-RBD antibodies compared to naive at baseline (*P* = 0.022), but this effect was not retained at postvaccination timepoints; no differences were detected in Spike-ACE2 inhibition activity between experienced and naive in LP-PWH (Fig. 4a).

(a) Anti-RDB antibodies (AUC) and Spike-ACE2 binding inhibition activity (% Inhibition) in SARS-CoV-2–naive (N) and -experienced (EXP) LP-PWH (*n*_N_ = 10, *n*_EXP_ = 10) and HCWs (*n*_N_ = 10, *n*_EXP_ = 10) at T0, T1, and T2. (b) SARS-CoV-2–specific IFN-γ+ CD4 T cells in SARS-CoV-2-naive (N) and -experienced (EXP) LP-PWH (*n*_N_ = 10, *n*_EXP_ = 10) and HCWs (*n*_N_ = 10, *n*_EXP_ = 10) at T0, T1, and T2. *Red/blue dots*: individual values; *black bars*: medians; *analyses*: Mann–Whitney *U* test (*P* values in black).

As for T-cell responses, SARS-CoV-2–experienced HCWs displayed higher S-specific IFN-γ+ CD4^+^ T cells at both T0 (*P* = 0.0251) and T1 (*P* = 0.0325), but not at T2. On the contrary, LP-PWH with prior SARS-CoV-2 infection did not show greater frequencies of S-specific T cells neither before nor after vaccination as compared to naive (Fig. 4b). No differences were found in other SARS-CoV-2–specific T-cell populations between naive and experienced vaccinees (data not shown).

## Discussion

In this prospective, longitudinal study, we sought to comparatively assess the magnitude and the quality of T-cell and humoral responses to COVID-19 mRNA vaccination up to 6 months apart from the primary cycle in cART-treated late presenter PWH as compared to HIV-negative people. We also evaluated whether previous SARS-CoV-2 infection modulates vaccine-elicited immune responses in these populations.

Although several recent studies have addressed the question examined in this work, most of them showing that PWH with optimal immunologic response to cART mount immune responses to SARS-CoV-2 vaccines comparable to those of the HIV-negative counterparts [25–31], our study specifically focused on PWH identified late in infection, a condition which has been reported to associate with enduring immune disfunction despite cART [43–46], thus raising the concern of suboptimal vaccine-elicited responses. Moreover, given that late presenters are at increased risk of poor immunological recovery [49,51,52,56,61,66], a state which has been described to be potentially linked with worse COVID-19 outcomes [8,14], it is of paramount importance to define the efficiency of immune responses to SARS-CoV-2 vaccines in such vulnerable population.

In our research, mRNA vaccine was able to expand SARS-CoV-2–specific T cells in a similar fashion in LP-PWH and HIV-uninfected people, with some minor phenotypic differences. CM, EM, and cTfh CD4^+^ T cells increased in both LP-PWH and HCWs, whereas S-specific EMRA CD4^+^ T cells only expanded in LP-PWH. Antigen-specific CD4 EMRA T cells have been reported to produce more IFN-γ compared to other memory subsets and to be endowed with potent cytotoxic effector functions, therefore being implicated in protective immunity against viral pathogens such as dengue virus (DENV) [67–69]. The observation that DENV–specific CD4 EMRA T cells expand with repeated infections [69] suggests that a high-dose/repeated antigen exposure may be critical for the development of such cells. Therefore, the induction of potentially protective SARS-CoV-2–specific CD4^+^ EMRA T cells in LP-PWH but not in HCWs may be explained with the different mRNA vaccine administered to the two groups, as mRNA-1273 has been proven to elicit stronger immune responses and to confer higher clinical protection compared to BNT162b2, possibly as a reflection of the 3.3-fold higher dose of mRNA [25,28,70–76].

Furthermore, – in accordance with prior data in HIV-negative vaccinees [59,77–80] – HCWs only spread SARS-CoV-2–specific Th1 cells, while LP-PWH developed vaccine-induced Th1 but also Th2-like (IL-4+) cells. A predominance of Th2 immune responses has long been known as main feature of HIV infection [81–84]. Furthermore, skewed T-cell responses to vaccines – including those against SARS-CoV-2 – with a predominant Th2 polarization have been described to associate with the aging-related immunosenescence [85–89]. This evidence may explain why in our study PWH, whose immune system is notoriously senescent [90], developed also Th2-like responses to vaccine.

Unexpectedly, frequencies of S-specific Th1 cells appeared to be higher in LP-PWH. However, iMFI for such cytokine-producing T cells were comparable between the two study groups, suggesting no significant differences in the total functional T-cell response to vaccine. Indeed, while the frequency of antigen-specific T cells only evaluates the magnitude of T-cell responses, iMFI is a metric which incorporates both the magnitude and quality of the immune response, thus reflecting the total functional response of a population of cytokine-producing T cells, so that it can be used to better estimate vaccine-induced protection [65,91,92]. Furthermore, polyfunctionality patterns of S-specific Th1 cells were similar in LP-PWH and HCWs, reaffirming that vaccine-induced T-cell responses are qualitatively comparable in the two study groups. In accordance with published data on SARS-CoV-2-specific T cells in both convalescent and vaccinated individuals [15,33,58,70,71,77–80], vaccine-induced CD4^+^ T-cell responses in our cohort outnumbered CD8^+^ T-cell responses.

We also found that mRNA vaccines induced spike-blocking anti-RBD antibodies in all vaccinees, irrespective of HIV status. Surprisingly, anti-RBD antibodies and spike-ACE2 binding inhibition activity, while comparable in the two groups at T1, were lower in HCWs at T2, possibly reflecting a faster decline of humoral responses to vaccine. As for the higher frequencies of vaccine-elicited CD4^+^ EMRA T cells in LP-PWH, the different mRNA vaccine administered to the two groups may explain such finding.

In agreement with previous studies which described antibody levels to wane more rapidly than T cells after both natural infection [93] and vaccination [59,70,94], we observed different dynamics in T-cell and humoral responses to mRNA vaccines, with a tendency to be stable or increase at 6 months in SARS-CoV-2-specific T cells and a tendency to decline in Spike-blocking anti-RBD antibodies in both LP-PWH and HCWs.

In accordance with previous studies in PWH demonstrating the association between immune response to SARS-CoV-2 mRNA vaccines and current CD4^+^ T-cell count [25–31,95–97], we hereby found a positive correlation between markers of immune recovery on cART and both SARS-CoV-2–specific CD8^+^ T-cells and antibody spike-ACE2 binding inhibition activity. Interestingly, a positive correlation between CD4^+^ T-cell *nadir* and spike-blocking function was also observed. These data suggest that PWH with low CD4^+^ T-cell *nadir* and/or poor immune reconstitution may have suboptimal responses to a two-dose vaccine cycle, reinforcing the importance of additional mRNA vaccine doses in these subgroups of PWH.

It should also be noted that LP-PWH at baseline showed lower percentages of naïve and higher frequencies of effector memory CD4^+^ and CD8^+^ T cells, as well as higher circulating T follicular cytotoxic cells. Depletion of naive T cells – which are critical for effective immune responses to pathogens and vaccines – is a well known feature of HIV infection [98]. Likewise, cTfc cells have been previously reported to be enriched in PWH and proposed as an indicator of disease progression because, among PWH, those with lower frequencies of cTfc cells also have lower CD4^+^ T-cell counts [99,100]. Nonetheless, in our cohort of LP-PWH, only naïve CD4^+^ T-cell pools out of the different subsets showed a positive correlation with vaccine-elicited immune responses (i.e., spike-blocking activity).

Finally, when we assessed the immune response according to previous COVID-19 diagnosis, we found a unique pattern of S-specific T-lymphocyte response in LP-PWH, that was not captured in HIV-uninfected people. Indeed, SARS-CoV-2-experienced LP-PWH, while showing higher anti-RBD antibodies at baseline similar to HIV-negative individuals, did not feature raised S-specific T cells, suggesting similar humoral yet diminished T-cell responses to natural infection. Furthermore, although previously exposed to SARS-CoV-2, LP-PWH failed to raise T-cell response at the end of the first vaccine cycle compared to SARS-CoV-2-naive ones, as instead observed in HCWs, pointing to an inability of prior SARS-CoV-2 infection to boost immune responses to vaccine in LP-PWH. These observations may appear in partial contrast with two previous studies, which described comparable SARS-CoV-2-specific T-cell and humoral responses between HIV-positive and negative individuals after SARS-CoV-2 infection [15,16]; however, PWH of our cohort have a history of late HIV presentation and feature lower median CD4^+^ and CD4^+^/CD8^+^ ratio than those included in the abovementioned studies, which may be responsible for reduced T-cell responses to SARS-CoV-2 natural infection. However, in accordance with our data, other recent studies showed that, compared to HIV-negative peers, convalescent PWH while developing similar humoral response, show lower and more exhausted SARS-CoV-2-specific T cells [17–19].

Some limitations need to be acknowledged in our study. Firstly, the lack of a CD3 marker in the flow cytometry antibody panels for both AIM and ICS assay, and the definition of the cTfh subset by means of the solely CXCR5 expression. Secondly, the small sample size, which may hinder the generalizability of the observations. Thirdly, the lack of data regarding SARS-CoV-2 variants of concern (VOCs), especially Omicron, not yet emerged at the time the study was conceptualized, and which is now the most widespread all over the world [101]. Fourthly, the relatively short follow-up and the lack of data on responses to a third/fourth dose of vaccine. Furthermore, the time between previous COVID-19 diagnosis and study enrollment was unknown in both LP-PWH and HCWs, so timing differences between the two groups might have influenced the dissimilarity found in infection-induced immune responses. Additionally, the mRNA vaccines administered were different in the two study groups, as per the initial indication of the Italian Ministry of Health (1273-mRNA in LP-PWH and BNT162b2 in HCWs), and this may account for a trend towards higher immune responses in LP-PWH.

In conclusion, in our cohort of PWH with pre-cART advanced immunodeficiency and current full virologic control on long-term cART, we herein showed that a two-dose mRNA-1273 vaccine cycle is able to induce both polyfunctional SARS-CoV-2–specific memory T cells and anti-RBD Spike-blocking antibodies, which are still above prevaccine baseline levels at 6 months. Of note, immune responses to vaccine do not appear inferior to those in HIV-negative peers overall, albeit a scarce immune recovery may hinder both T-cell and humoral vaccine-elicited responses. Furthermore, unlike HIV-negative individuals, in LP-PWH natural SARS-CoV-2 infection seems inefficient in inducing specific T-cell memory and in boosting T-cell and humoral responses to vaccine, reflecting an enduring partial immune dysfunction. Altogether, these findings support the need for additional vaccine doses in PWH with a history of advanced immune depression and poor immune recovery on effective cART.

## Acknowledgements

We are grateful to all the people enrolled in this study who agreed to participate to this research. Our special thanks also go to all the physicians and nurses at the Clinic of Infectious Diseases and Tropical Medicine at San Paolo Hospital in Milan who continuously help in patients’ care and vaccination management, as well as laboratory personnel whose role in the conduct of the study was crucial.

Author contributions: M.A. contributed to study design, enrolled participants, collected clinical data, designed and performed the experiments, analyzed and interpreted the data, designed the figures, and wrote the manuscript; V.B. and R.R. designed and performed the experiments and interpreted the data; C.T. contributed to study design, data interpretation and critical revision of the manuscript; A.d’A.M. contributed to critical revision of the manuscript; G.M. conceived and designed the study, interpreted the data, and wrote the manuscript; all authors contributed to the article and approved the submitted version.

Funding: This work was supported by grants from Fondazione Cariplo in collaboration with Regione Lombardia and Fondazione Umberto Veronesi (CAR_RIC20GMARC_01 and CAR_RIC20GMARC_02) and by Fondazione di Comunità Milano (FON_NAZ20ADARM_01).

### Conflicts of interest

There are no conflicts of interest.

*This study was presented in part at HIV Glasgow 2022 (#P248).*

## Supplementary Material

Supplemental Digital Content
