# Supplementary material for: Six-month immune responses to mRNA-1273 vaccine in combination antiretroviral therapy treated late presenter people with HIV according to previous SARS-CoV-2 infection
Source: AIDS. 2023 May 18;37(10):1503–17. doi: 10.1097/QAD.0000000000003585 (PMC10355808; doi:10.1097/QAD.0000000000003585)
Supplement: Supplemental Digital Content [file aids-37-1503-s001.pdf]

Supplementary material

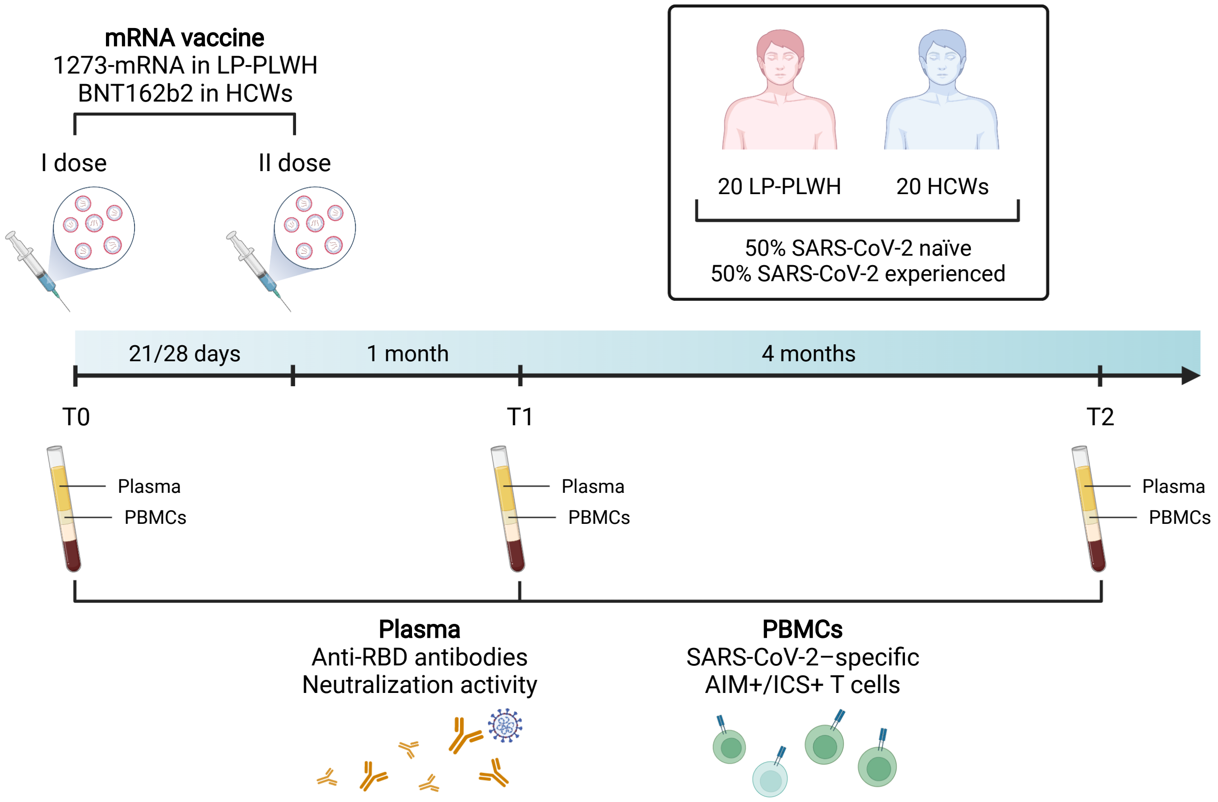

Figure S1. Study design. Scheme representing the study design. T0: before vaccination; T1: 1 month after the second dose; T2: 5 months after the second dose (6 months after the first dose). Created with *BioRender.com*.

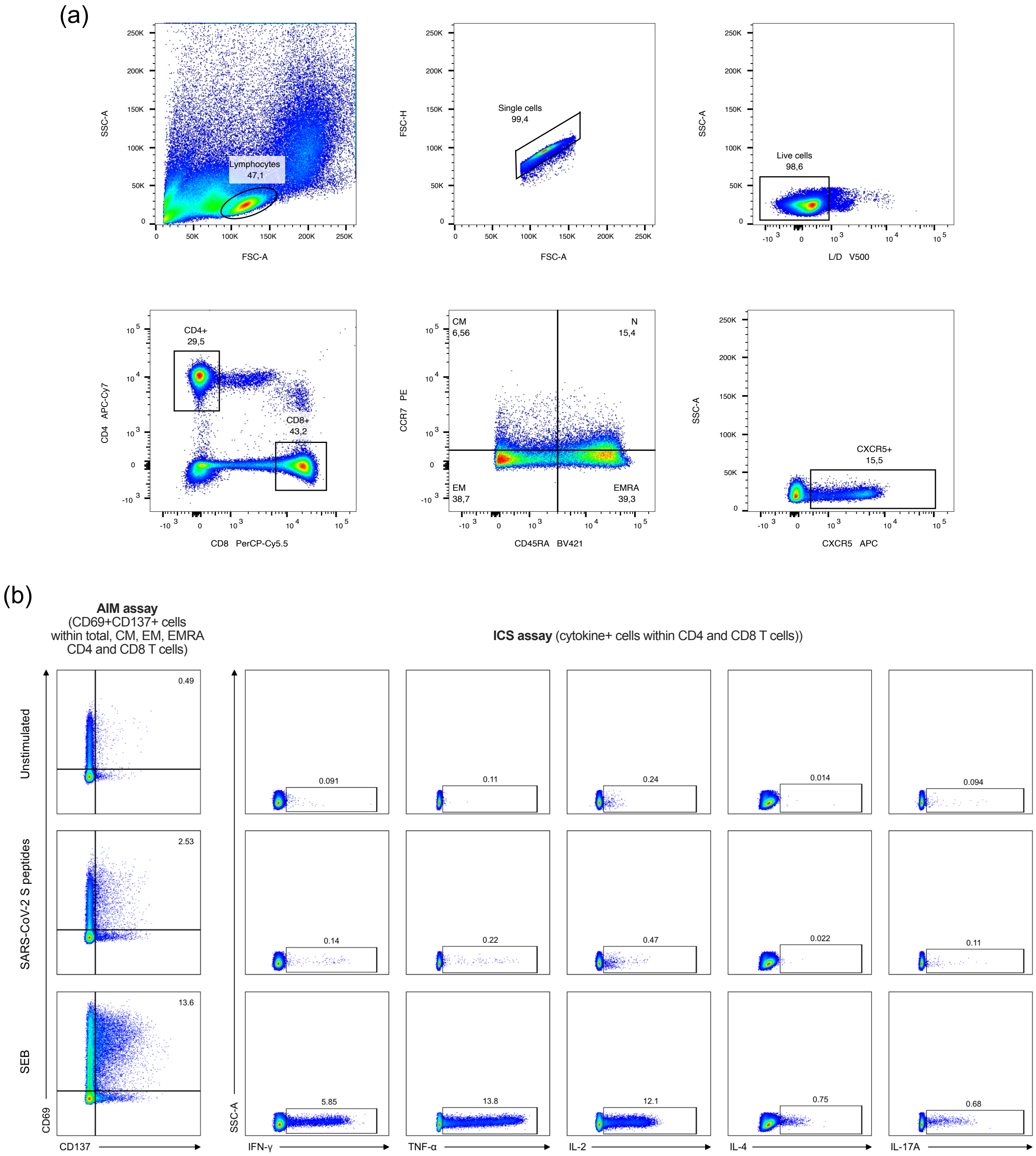

Figure S2. Flow cytometry gating strategy and assays for SARS-CoV-2–specific T-cells detection. (a) Lymphocytes, single and live cells were identified, and further divided into CD4+ and CD8+ cells. Maturation subsets (according to CCR7 and CD45RA expression) and CXCR5+ cells were then identified within CD4 and CD8 T-cells. (b) Flow cytometry gating strategy used for the measurement of AIM+ (CD69+CD137+) and ICS+ (IFN-γ+, TNF-α+, IL-2+, IL-4+, IL-17A+) CD4 and CD8 T-cells in PBMCs unstimulated, stimulated with wild-type SARS-CoV-2 Spike protein peptides pool, and stimulated with *Staphylococcus* enterotoxin B (SEB). AIM+ cells were determined within total, CM, EM, and EMRA CD4 and CD8 T-cells, whilst ICS+ cells within CD4 and CD8 T-cells. SARS-CoV-2–specific T-cells were measured subtracting unspecific background activation (AIM) or cytokine-production (ICS) in unstimulated samples from samples stimulated with SARS-CoV-2 Spike protein peptides pool; samples stimulated with SEB were used as positive controls.

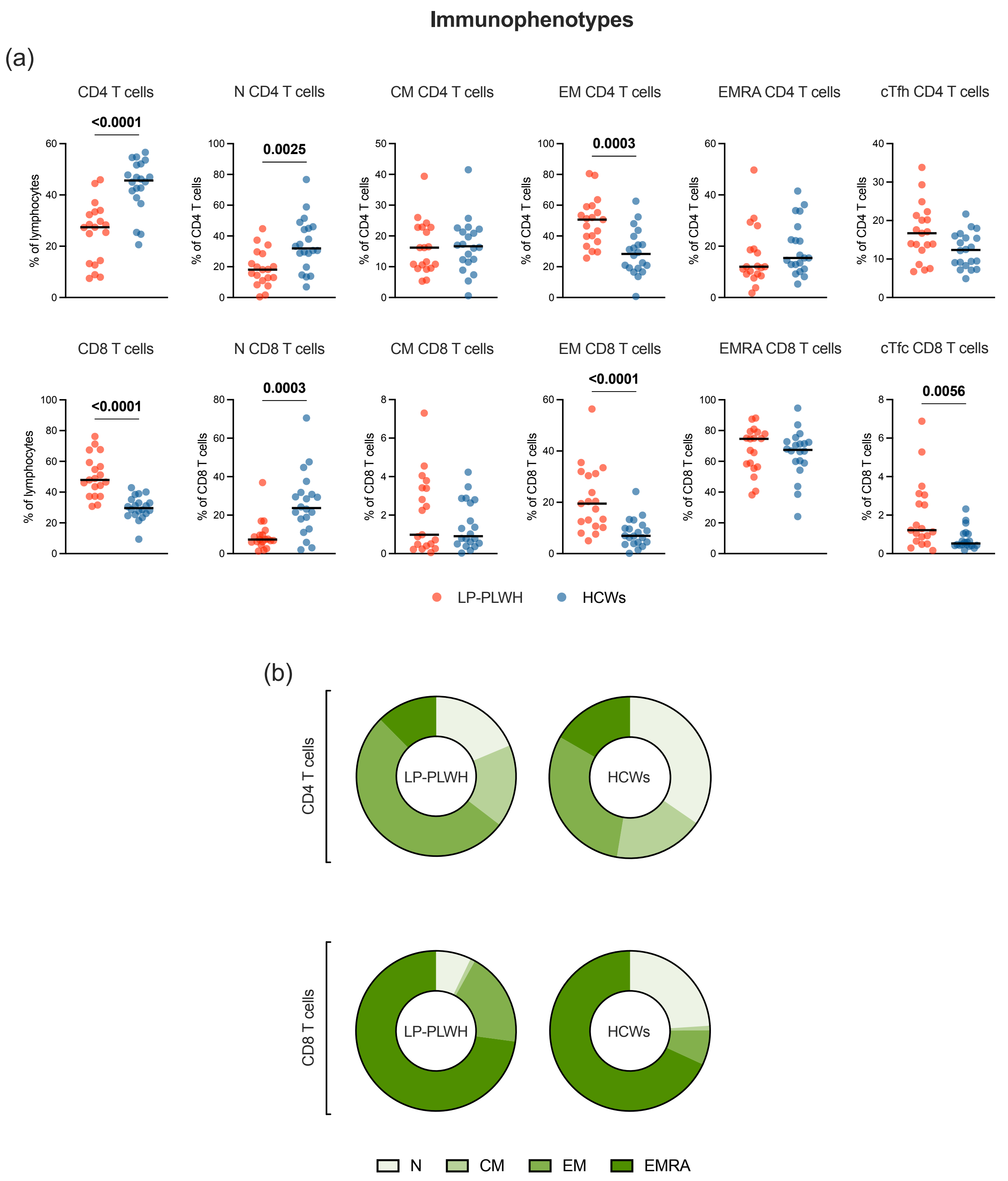

Figure S3. Immunophenotypes at baseline. T-cell immunophenotypes at baseline (T0) in LP-PLWH (*n*=19) and HCWs (*n*=20): total, naïve (N, CCR7+CD45RA+), central memory (CM, CCR7+CD45RA–), effector memory (EM, CCR7–CD45RA–), effector memory re-expressing CD45RA (EMRA, CCR7–CD45RA+) CD4 and CD8 T-cells as well as circulating T follicular helper (cTfh, CD4+CXCR5+) and circulating T follicular cytotoxic (cTfc, CD8+CXCR5+) cells. *Red/blue dots*: individual values; *black bars*: medians; *analyses*: Mann-Whitney U test (*P* values in black). **(c)** Median distribution of N, CM, EM, and EMRA phenotypes within CD4 and CD8 T-cells in LP-PLWH and HCWs are shown in donut charts as the median percentage (donut slice) of total CD4 and CD8 T-cells.

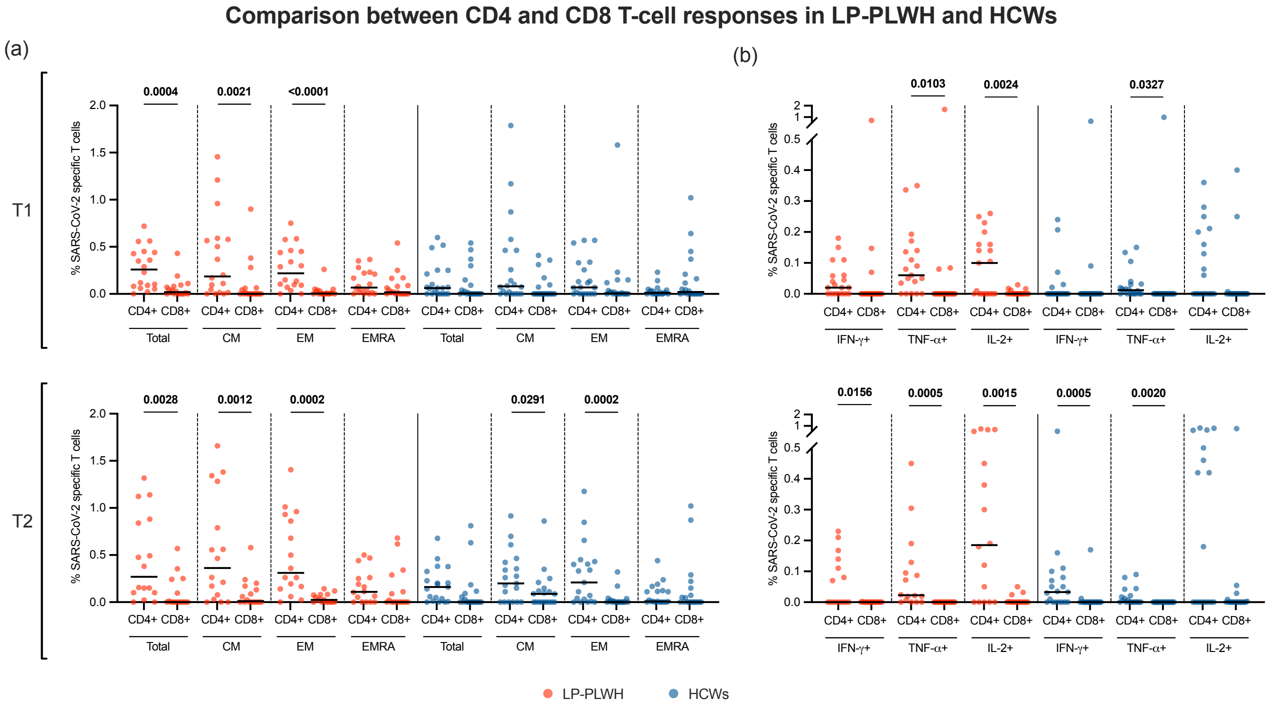

Figure S4. Comparison between vaccine-induced SARS-CoV-2–specific CD4 and CD8 T-cell responses. Frequencies (percentage, %) of SARS-CoV-2–specific AIM+ and ICS+ CD4 and CD8 T-cells in LP-PLWH and HCWs at T1 and T2. *Red/blue dots*: individual values; *black bars*: medians; *analyses*: Mann-Whitney U test (*P* values in black).
